# Romosozumab after rebound vertebral fractures induced by denosumab discontinuation: a case series

**DOI:** 10.1093/jbmrpl/ziaf186

**Published:** 2025-12-08

**Authors:** Marcelo Sarli, Vanina Farias, Rubén Abdala, Estefanía Armanelli, Cintia Bocci, Fernando Jerkovich, María Belén Zanchetta

**Affiliations:** Department of Endocrinology, IDIM, Buenos Aires, C1012AAR, Argentina; Department of Endocrinology, IDIM, Buenos Aires, C1012AAR, Argentina; Department of Endocrinology, IDIM, Buenos Aires, C1012AAR, Argentina; Department of Bone and Mineral Metabolism, Facultad de Medicina, Universidad del Salvador, Buenos Aires, C1055AAG, Argentina; Department of Bone and Mineral Metabolism, Facultad de Medicina, Universidad del Salvador, Buenos Aires, C1055AAG, Argentina; Department of Endocrinology, IDIM, Buenos Aires, C1012AAR, Argentina; Department of Endocrinology, Hospital General de Agudos Dr. Juan A. Fernández, Buenos Aires, C1425AGP, Argentina; Department of Endocrinology, IDIM, Buenos Aires, C1012AAR, Argentina

**Keywords:** anabolics, antiresorptives, osteoporosis, biochemical markers of bone turnover, DXA, bone QCT/micro-CT

## Abstract

Introduction: Discontinuation of denosumab can lead to a rebound increase in bone resorption, which may result in multiple vertebral fractures. Current guidelines recommend retreatment with denosumab or administration of zoledronate in this setting. Materials and methods: We report a series of 4 patients who developed vertebral fractures following denosumab withdrawal and were subsequently treated with romosozumab, a dual-action agent with anabolic and antiresorptive properties. Results: All patients showed progressive improvement in BMD and microarchitecture, with suppression of bone turnover markers and no new clinical fractures during treatment and follow-up. Discussion: Although limited by the uncontrolled design and small sample size, these observations suggest that romosozumab might be considered as a potential therapeutic approach for selected patients with post-denosumab rebound fractures.

## Graphical Abstract

**Graphical Abstract:**
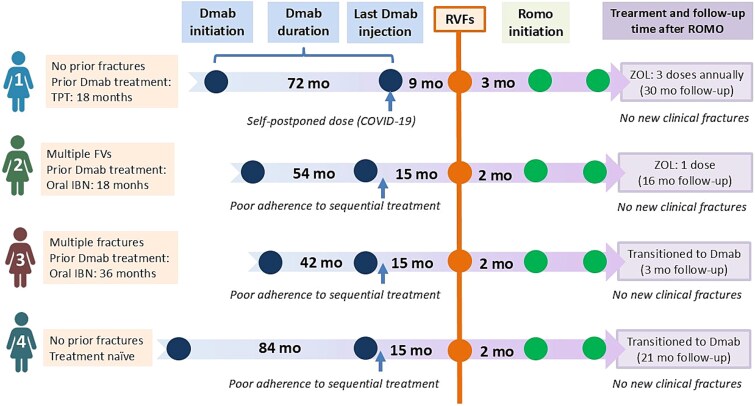

## Introduction

Denosumab, a monoclonal antibody targeting RANKL, has demonstrated significant efficacy in preventing fragility fractures. However, due to its reversible mechanism of action, sequential treatment with antiresorptives is recommended to counteract the increase in bone remodeling following discontinuation and to mitigate the risk of rebound vertebral fractures (RVFs).^1^^,^^2^ This phenomenon represents one of the most serious complications faced by clinicians when planning denosumab discontinuation, and there are currently no randomized controlled trials specifically designed to address optimal management strategies in this setting.

To mitigate this risk, sequential treatment with bisphosphonates following denosumab cessation is recommended.^2^ However, in patients who develop RVFs despite preventive measures or in whom bisphosphonates were not administered, international guidelines suggest either resuming denosumab or initiating a potent antiresorptive agent, such as zoledronate, as first-line options.^3^ While these approaches effectively suppress bone resorption, they do not provide an anabolic stimulus to rebuild compromised bone structure.

Romosozumab, a monoclonal antibody targeting sclerostin, exerts a dual effect by stimulating bone formation while simultaneously inhibiting bone resorption.^4^ This unique mechanism raises the hypothesis that romosozumab could represent a valuable alternative for patients with post-denosumab RVFs, potentially offering both structural restoration and prevention of further bone loss. Notably, a recent publication demonstrated that the use of romosozumab following denosumab did not produce rebound resorption nor attenuate the anabolic effect of romosozumab,^5^ suggesting that this therapeutic sequence may be feasible. However, clinical evidence supporting the use of romosozumab in the specific context of post-denosumab RVFs remains limited.

This case series describes 4 patients who developed RVFs after denosumab discontinuation and were subsequently treated with romosozumab. We analyzed their densitometric response by DXA, changes in bone turnover markers (BTMs), microarchitectural parameters assessed by HR-pQCT, and the incidence of new clinical fractures during follow-up. Despite the small sample size, this study represents a first step toward exploring romosozumab as a therapeutic alternative in this challenging clinical scenario, with potential implications for optimizing sequential osteoporosis treatment strategies.

## Patients

This study includes 4 patients who had been treated with denosumab and subsequently developed RVFs after its discontinuation. Following the RVFs, all patients received romosozumab for 1 yr. Detailed individual case descriptions are provided in the “Results” section. Informed consent was obtained from all patients, and approval for publication was granted by the institution’s academic committee.

## Materials and methods

Data were retrospectively collected from electronic medical records, encompassing clinical, densitometric (DXA), laboratory, and bone microarchitecture information.

Radiological evaluation, including lateral X-ray of the thoracic and LS, was performed at baseline (at the time of RVF diagnosis) and at the end of ROMO therapy (12 mo) for all patients. During follow-up, repeat imaging was performed if symptoms suggestive of new fractures appeared (eg, acute back pain, height loss). BMD was assessed by DXA using a GE Lunar Prodigy scanner (GE Lunar), and the results were reported in g/cm^2^ and T-score (TS). The sites evaluated included the LS, FN, and TH. In one patient, due to bilateral hip replacement, the 33% distal radius (DR) was measured pre- and post-romosozumab treatment. The equipment was calibrated daily according to the manufacturer’s instructions. Precision studies conducted at our center showed inter-assay coefficients of variation of 1.53% for the LS, 1.68% for the FN and TH, 1.34% for the 33% radius, and 1.14% for whole-body measurements. The least significant change (LSC) at 95% CI was calculated as LSC = 2.77 × CV%, yielding thresholds of 4.24% for LS, 4.65% for TH, and 3.71% for DR.

Bone microarchitecture was evaluated using HR-pQCT (XtremeCT, Scanco Medical AG) of the non-dominant distal tibia and DR, performed before and after romosozumab treatment. The scan generates a 3D representation from 110 slices with a voxel size of 82 μm, acquired at 22.5 mm (tibia) and 9.5 mm (radius) from a reference line at the end plate. At both sites, the following parameters were assessed: total volumetric BMD (vBMD), trabecular vBMD, cortical vBMD, trabecular number, trabecular thickness, trabecular separation, trabecular heterogeneity, and cortical thickness. The first 3 are considered densitometric parameters, while the remaining are classified as structural parameters.

Laboratory analyses included BTMs measured before and after romosozumab treatment. Blood samples were collected in the fasting state before 10 am to minimize circadian variation. BTMs assessed included: bone-specific alkaline phosphatase (BAP; chemiluminescence method, Liaison XL Assay, Diasorin Inc.; normal value < 21.3 μg/L), β-CrossLaps (CTX; electrochemiluminescence method, Elecsys assay, Cobas e-411, Roche Diagnostics GmbH; reference range for postmenopausal women: 556 ± 226 pg/mL), and osteocalcin (BGP; electrochemiluminescence method, Elecsys assay, Cobas e-411, Roche Diagnostics; reference range: 11-43 ng/mL).

Additionally, data were collected on total follow-up duration after romosozumab treatment, subsequent osteoporosis therapies, and any new clinical fractures occurring during follow-up.

## Results

**Figure 1:**
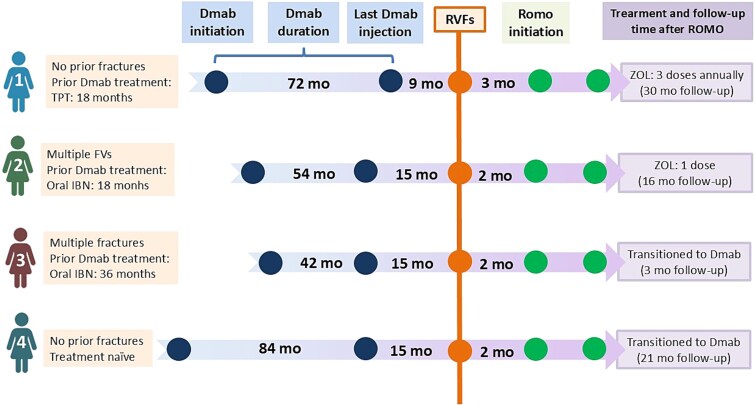
Schematic treatment timelines for 4 patients with rebound vertebral fractures (RVFs) after denosumab discontinuation. Timeline representation (not to scale) showing treatment sequences from denosumab initiation through post-romosozumab follow-up. Orange circles with vertical line indicate rebound vertebral fracture (RVF) events. Green circles mark romosozumab treatment initiation and completion (12 mo). Numbers represent time intervals in months (mo). Patient characteristics and prior treatments are shown on the left. Abbreviations: Dmab, denosumab; IBN, ibandronate; ROMO, romosozumab; RVFs, rebound vertebral fractures; TPT, teriparatide; ZOL, zoledronic acid.

**Figure 2:**
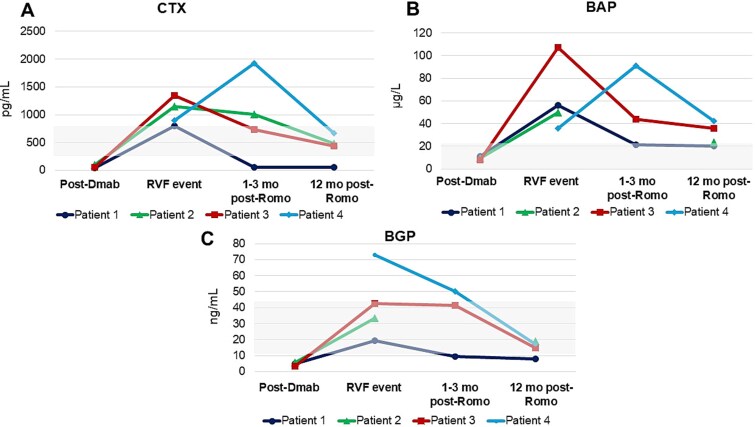
Biochemical markers of bone turnover (BTMs) over time: (A) CTX, (B) BAP, and (C) BGP measured at baseline (post-denosumab, prior to discontinuation), at the time of rebound vertebral fractures (RVFs), at 1-3 mo, and at 12 mo after romosozumab initiation. In Patient 1, the early post-romosozumab assessment was performed at 1 mo, while in the remaining patients it was performed at 3 mo. Abbreviations: BAP, bone-specific alkaline phosphatase; BGP, osteocalcin; CTX, β-CrossLaps; Dmab, denosumab; Romo, romosozumab; RVFs, rebound vertebral fractures.

This case series included 4 patients who had received denosumab for varying durations (range: 3.5-7 yr) prior to discontinuation. In Patient 1, the scheduled denosumab dose was postponed by patient choice in the context of the COVID-19 pandemic, whereas in Patients 2-4 discontinuation occurred due to inadequate adherence to recommended sequential therapy. The time interval from last denosumab dose to RVF diagnosis ranged from 9 to 15 mo. The baseline characteristics and prior osteoporosis treatments are summarized in Table 1. A schematic timeline of denosumab duration, timing of RVFs, and initiation of romosozumab for each patient is shown in Figure 1. Changes in areal BMD over 12 mo of romosozumab treatment are reported in Table 2, and HR-pQCT microarchitectural parameters are presented in Table 3. The evolution of BTMs over time is displayed in Figure 2.

**Table 1:** Clinical characteristics and prior osteoporosis treatment before romosozumab initiation.

**Characteristics**	**Patient 1**	**Patient 2**	**Patient 3**	**Patient 4**
Age (yr)	61	75	70	64
Height (m)	1.62	1.56	1.66	1.66
Weight (kg)	63	63	55	71
BMI (kg/m^2^)	24	29	20	26
Age at menopause	50	40	48	51
Number of prior fragility fracture(s)	-	5	6	-
10-yr hip fracture risk (FRAX) (%)^a^	7	18	11	13
10-yr MOF risk (FRAX) (%)	15	26	19	7
Osteoporosis treatment prior to denosumab	Teriparatide (18 mo)	Ibandronate (36 mo)	Ibandronate (60 mo)	None
Duration of denosumab treatment (mo)	72	54	42	84
Time from last denosumab injection to RVF event (mo)	9	15	15	15
Number of RVFs	5	5	6	4

a FRAX 10-yr probabilities of major osteoporotic and hip fracture were calculated using the Argentina country model. For most patients, FN BMD values obtained from DXA scans (GE Lunar Prodigy, GE Lunar) were incorporated. In one patient (Case 3), FRAX was calculated based on clinical risk factors only, as hip BMD could not be measured due to bilateral hip fractures.

Abbreviations: FRAX, Fracture Risk Assessment Tool; MOF, major osteoporotic fracture; RVFs, rebound vertebral fractures.

**Table 2:** Changes in BMD during prior denosumab therapy and after 12-mo romosozumab treatment.

**Patient**	**Site**	**Δ BMD on Dmab**	**Baseline BMD (g/cm** ^**2**^**)**	**Baseline T-score**	**12-mo BMD (g/cm** ^**2**^**)**	**12-mo T score**	**Δ BMD on ROMO**	**Exceeds LSC?** ^a^
1	LS	+2.9%	0.930	−2.1	0.944	−2.0	+1.5%	No
1	TH	+8.5%	0.707	−2.4	0.668	−2.8	−5.5%	Yes^b^
2	TH	−7.5%	0.717	−1.5	0.759	−2.0	+5.8%	Yes
3	LS	+8.3%	0.929	−2.1	1.262	+0.5	+35.8%	Yes
3	33% DR	-	0.641	−2.7	0.678	−2.3	+5.8%	Yes
4	LS	+10. 3%	0.780	−3.5	0.833	−3.1	+6.8%	Yes
4	TH	+6.1%	0.598	−3.3	0.661	−2.8	+10.5%	Yes

a Least significant change (LSC) thresholds for ROMO response: LS = 4.2%, TH = 4.6%, and DR = 3.7%.

b Negative change exceeding LSC (bone loss during ROMO treatment).

Abbreviations: BMD: bone mineral density; LS: lumbar spine; TH: total hip; DR, distal radius (33%).

**Table 3:** HR-pQCT parameters before and after romosozumab treatment.

**Patient**	**HR-pQCT parameter**	**Radius**			**Tibia**		
		**Baseline**	**12-mo**	**Δ HR-pQCT**	**Baseline**	**12-mo**	**Δ HR-pQCT**
1	Ct.iBMD (mg HA/cm^3^)	198.8	193.5	−2.1%	180.6	174.8	−3.2%
	Ct.vBMD (mg HA/cm^3^)	741.4	731.3	−1.4%	764.1	741.2	−3.0%
	Ct.Th (mm)	0.54	0.48	−11.1%	0.54	0.48	−11.1%
	Tb.vBMD (mgHA/cm^3^)	113.7	113.7	0%	120.2	120.9	+0.6%
	Tb.BVTV (%)	9.5	9.5	0%	10.0	10.1	+1.0%
	Tb.N. 1/mm	1.63	1.69	+3.7%	1.70	1.82	+7.4%
	Tb.Th. mm	0.059	0.055	−6.4%	0.059	0.055	−6.4%
2	Ct.iBMD (mg HA/cm^3^)	241.2	328.8	+36.3%	176.4	185.8	+5.3%
	Ct.vBMD (mg HA/cm^3^)	850.1	826.1	−2.8%	648.7	719.4	+10.9%
	Ct.Th (mm)	0.63	0.87	+38.1%	0.34	0.46	+36.8%
	Tb.vBMD (mgHA/cm^3^)	86.2	148.9	+72.7%	131.6	131.4	−0.1%
	Tb.BVTV (%)	7.2	12.4	+72.2%	11.0	11.0	0%
	Tb.N. 1/mm	1.10	1.11	+0.9%	2.04	1.66	−19.0%
	Tb.Th. mm	0.065	0.112	+71.1%	0.054	0.066	+22.9%
3	Ct.iBMD (mg HA/cm^3^)	202.6	193.9	−4.3%	185.1	178.9	−3.3%
	Ct.vBMD (mg HA/cm^3^)	843.8	812.4	−3.8%	717.2	720.3	+0.4%
	Ct.Th (mm)	0.54	0.48	−11.1%	0.49	0.48	−2.0%
	Tb.vBMD (mgHA/cm^3^)	81.0	84.8	+4.7%	128.7	123.6	−4.0%
	Tb.BVTV (%)	6.8	7.1	+4.4%	10.7	10.3	−3.7%
	Tb.N. 1/mm	1.12	1.11	−0.7%	2.02	1.57	−0.2%
	Tb.Th. mm	0.060	0.064	+5.4%	0.053	0.066	+24.0%
4	Ct.iBMD (mg HA/cm^3^)	209.1	195.3	−6.6%	162.1	168.8	+4.1%
	Ct.vBMD (mg HA/cm^3^)	841.6	825.9	−1.9%	773.9	789.4	+2.0%
	Ct.Th (mm)	0.54	0.50	−7.4%	0.62	0.68	+9.7%
	Tb.vBMD (mgHA/cm^3^)	78.5	73.8	−6.0%	83.2	86.8	+4.3%
	Tb.BVTV (%)	6.5	6.1	−6.0%	6.9	7.2	+4.3%
	Tb.N. 1/mm	0.97	1.00	+2.6%	0.96	1.03	+7.6%
	Tb.Th. mm	0.067	0.062	−8.3%	0.072	0.070	−3.1%

Abbreviations: Ct.iBMD, integral cortical density; Ct.vBMD, cortical volumetric BMD; Ct.Th, cortical thickness; Tb.BVTV, trabecular bone volume fraction; Tb.N, trabecular number; Tb.Th, trabecular thickness; Tb.vBMD, trabecular density.

ROMO was chosen over guideline-recommended options based on individualized clinical considerations: Patient 1 had documented non-adherence to denosumab, necessitating treatment rotation; Patient 2 experienced progressive hip BMD loss despite denosumab, suggesting need for a more potent therapy; Patient 3 declined denosumab resumption; and Patient 4 had severe microarchitectural deterioration warranting anabolic intervention. In all cases, patient preference and clinical judgment favored an anabolic approach to rebuild bone structure, and no cardiovascular contraindications to ROMO were present.

The follow-up periods after completion of romosozumab therapy were 30, 16, 3, and 21 mo, respectively. Two patients received sequential treatment with zoledronic acid (5 mg annually), and 2 of them received denosumab (60 mg every 6 mo) (Figure 1).

No new fragility fractures, including clinical or morphometric vertebral fractures, were observed during romosozumab treatment or throughout the subsequent follow-up period.

### Patient 1

A 61-yr-old woman with no prior fragility fractures and very low BMD was initially treated with teriparatide for 18 mo, followed by 6 yr of denosumab. Her BMD improved substantially at the LS and TH during sequential therapy (Table 2). In 2021, after postponing a scheduled denosumab dose during the COVID-19 pandemic, she developed acute low back pain following a minor fall. MRI revealed multiple acute vertebral fractures (T7, L1, L2, L3, and L5), and laboratory tests showed a marked rebound in BTMs (CTX: 800 pg/mL, BGP: 56.8 ng/mL, and BAP: 19.4 μg/L), consistent with RVFs.

Romosozumab treatment was initiated in November 2021. One month later, BTMs decreased (CTX: 58 pg/mL, BGP: 21.5 ng/mL, and BAP: 9.3 μg/L). HR-pQCT at baseline revealed low volumetric BMD at both the radius and tibia, with notable cortical deterioration. After 12 mo of romosozumab, there were no changes in cortical parameters, but a 3.7% increase in trabecular number was observed in the radius. Areal BMD stabilized at the LS but decreased by 5.5% at the TH (Table 2).

In October 2022, she transitioned to antiresorptive therapy with annual zoledronic acid (5 mg i.v.). After 3 consecutive annual doses (36 mo of follow-up), TH BMD increased by 6.6% compared to post-romosozumab values, while LS BMD decreased by 7%. No new clinical fractures have occurred since initiating romosozumab.

### Patient 2

A 75-yr-old woman with a history of L1-L2 vertebral fractures (treated with vertebroplasty), nephrectomy for tuberculosis, chronic inhaled corticosteroid use, and a maternal history of hip fracture was referred for osteoporosis management. She had previously received ibandronate for 3 yr with no DXA improvement. DXA showed low BMD, and CTX was elevated (848 pg/mL). Denosumab was initiated and maintained for 4.5 yr, with stable BMD and suppressed BTMs (CTX 71 pg/mL). Discontinuation was followed by a brief course of alendronate, which the patient discontinued after 1 mo.

Four months later, she developed acute thoracolumbar pain, and imaging confirmed 5 recent vertebral fractures (T8, T10, T12, L3, and L4), with elevated BTMs (CTX 1150 pg/mL), consistent with RVFs. Romosozumab was started in August 2022. CTX decreased progressively during treatment (to 1004, 460, and 447 pg/mL at 3, 6, and 12 mo, respectively). DXA performed after 12 mo of treatment showed a 6% increase in TH BMD (Table 2). HR-pQCT revealed improvements in cortical and trabecular microarchitecture in the radius and cortical bone in the tibia (Table 3).

She transitioned to zoledronic acid (5 mg i.v.), with further reduction in CTX 1 mo post-infusion. At 16 mo of follow-up, she remains free of new clinical fractures.

### Patient 3

A 70-yr-old woman with a long-standing diagnosis of osteoporosis and multiple fragility fractures—including bilateral hip fractures (at ages 48 and 70) and a right wrist fracture—was referred after sustaining a left hip fracture that required TH replacement. She had previously received ibandronate for 5 yr with poor adherence to both treatment and follow-up. DXA in 2018 showed low LS BMD (0.906 g/cm^2^, T-score −2.3), and BTMs were elevated (CTX 631 pg/mL). Denosumab was administered regularly for 7 doses. After treatment, spine BMD increased by 8%, and BTMs were suppressed (CTX 54 pg/mL).

She discontinued denosumab and medical follow-up in December 2021. Fifteen months later, she returned with acute low back pain. Imaging revealed multiple recent vertebral fractures (L5 on MRI; D5, D6, D9, D12, and L1 on VFA) and markedly elevated BTMs (CTX: 1341 pg/mL, BAP: 42.5 μg/L, BGP: 107 ng/mL), consistent with RVFs.

Given her refusal to resume denosumab, romosozumab was initiated in July 2023. CTX decreased gradually (to 442 pg/mL at 12 mo). DXA in June 2024 showed a 36% increase in LS BMD and a 6% increase at the 33% DR. HR-pQCT confirmed trabecular gains in the radius.

She completed 12 mo of romosozumab without new fractures, and then transitioned to denosumab, which she continues to receive.

### Patient 4

A 64-yr-old woman with a history of primary hyperparathyroidism, treated with parathyroidectomy in 2014 (parathyroid adenoma) with complete remission, subsequently initiated denosumab due to markedly low bone mass without prior fractures. She received the drug for 7 yr with an excellent response. In December 2022, denosumab was discontinued and switched to oral bisphosphonates; however, adherence was poor and limited to less than 3 mo, after which the patient discontinued therapy.

Ten months after denosumab discontinuation, she sustained a vertebral fracture following physical exertion, which required surgical fixation. At postoperative follow-up, multiple new vertebral fractures were detected, and romosozumab was initiated.

After 3 mo of romosozumab, serum CTX values had doubled. Although rotation to zoledronic acid was advised, the patient chose to continue with romosozumab. At 6 mo, CTX levels began to decline, ultimately reaching values below baseline by the end of the 12-mo course. DXA BMD increased 7% at the LS and 10% at the TH, although very low bone mass persisted (LS: TS −3.1 and TH: −2.8).

Given this context, denosumab was reinitiated. To date, she has received 4 doses without complications and has remained free of new clinical or morphometric fractures.

## Discussion

We report a case series of 4 patients who sustained RVFs and were subsequently treated with romosozumab, an approach that deviates from current recommendations. The European Calcified Tissue Society (ECTS) position statement, based on a systematic review of literature published until August 2020 and expert consensus, recommends either resuming denosumab therapy or administering zoledronic acid as first-line options for managing patients following denosumab discontinuation.^3^ However, these recommendations are derived from expert opinion rather than randomized controlled trials, as no RCTs specifically addressing management strategies for established RVFs have been published to date. To our knowledge, this is among the first published series describing the use of romosozumab specifically in patients with established RVFs following denosumab discontinuation. While 2 previous case reports described romosozumab use after denosumab withdrawal,^6^^,^^7^ our series is the first to systematically evaluate clinical outcomes, densitometric response, BTM suppression, and microarchitectural changes in patients with documented RVFs.

Although the cohort is heterogeneous, all patients were at high fracture risk. Across the four cases, we observed variable changes in areal BMD and trabecular microarchitecture. Notably, the modest antiresorptive action of romosozumab was sufficient to counteract the rebound in bone resorption, and its anabolic effect was preserved despite prior denosumab exposure. No new clinical fractures were reported during the year of treatment or throughout follow-up.

In the 2 previously published cases reported the use of romosozumab following denosumab discontinuation, both resulted in further fractures despite treatment.^6^^,^^7^ In the first case,^6^ romosozumab was initiated 9 mo after the last denosumab dose, at a time when BTMs (P1NP and TRACP-5b) were markedly elevated, and the patient subsequently developed 5 new vertebral fractures with no gain in LS BMD after 6 mo. In the second case,^7^ romosozumab was started 7 mo after the last denosumab injection in a patient with multiple prior treatment interruptions and a history of recurrent fractures during drug holidays. Bone turnover markers (CTX and P1NP) remained markedly elevated, and the patient experienced a cascade of 5 new vertebral fractures within 6 mo.

In contrast, some of our cases differ in 2 critical aspects: timing of intervention and magnitude of BTM elevation at romosozumab initiation. Romosozumab was initiated promptly, 2-3 mo after RVF diagnosis, likely before the full magnitude of the rebound phenomenon had occurred. Additionally, while BTMs were elevated at baseline in our patients, the degree of elevation may have been lower than in the previously reported cases. We hypothesize that both the timing of romosozumab initiation and the magnitude of BTM elevation at treatment start may be putative effect modifiers influencing treatment response. Early intervention may have allowed romosozumab’s modest antiresorptive effect to effectively counterbalance the evolving rebound in bone resorption, while its anabolic action rebuilt bone structure.

However, it is critical to emphasize that causality cannot be inferred from this uncontrolled, heterogeneous case series. The favorable outcomes observed may reflect not only the timing and pharmacological effects of romosozumab but also differences in baseline fracture risk, prior treatment histories, and subsequent consolidation therapy. Controlled studies are needed to definitively establish whether early romosozumab initiation improves outcomes in post-denosumab RVFs.

In our study, BTM trajectories showed variable patterns across patients. While 3 patients demonstrated rapid CTX suppression within 1-3 mo, Patient 4 exhibited a paradoxical transient increase at 3 mo before subsequent decline. This heterogeneity may reflect differences in the magnitude and timing of the rebound phenomenon, prior treatment adherence, and individual pharmacodynamic variability. Despite this variability, all patients ultimately achieved BTM suppression by 12 mo and remained fracture-free, suggesting that romosozumab’s dual mechanism can effectively counteract rebound resorption even when early BTM kinetics are atypical. These observations underscore the importance of individualized monitoring and the potential need for flexible treatment strategies in this challenging clinical scenario.

Recent clinical data provide additional context for romosozumab use after denosumab. Hong et al.^5^ showed that switching to romosozumab after 2 yr of denosumab led to greater gains in LS BMD and trabecular bone score compared to continued denosumab treatment. In another study, Adami et al.^8^ reported that adding romosozumab to ongoing denosumab therapy enhanced the anabolic window more effectively than sequential administration. However, no superiority over continued denosumab treatment was demonstrated—likely due to the limited sample size.

Our institution has previously investigated rebound bone loss after denosumab discontinuation. In a cohort without subsequent therapy, we reported substantial losses of BMD within 17 mo after stopping denosumab (LS: −8%, FN: −6%, and TH: −8%), accompanied by a 13% incidence of new vertebral fractures.^9^ In another study, patients who received bisphosphonates after denosumab withdrawal still experienced modest BMD losses over 36 mo (LS: −4%, FN: −2%, TH: −4%), indicating that sequential therapy attenuates but does not fully prevent bone loss.^10^ In contrast, in the present series, patients treated with romosozumab after denosumab withdrawal showed average BMD increases of 15% at the LS, 4% at the TH, and 6% at the DR over 12 mo. These findings are consistent with a potential protective effect of romosozumab on both trabecular- and cortical-rich bone, in contrast with the marked losses observed after denosumab discontinuation without subsequent therapy.

An important limitation in interpreting fracture outcomes is the inherent challenge of attributing the antifracture effect specifically to romosozumab in a sequential treatment context. Like all osteoanabolic therapies, romosozumab requires sequential antiresorptive therapy to consolidate skeletal gains and prevent bone loss. Therefore, it is difficult to determine how much of the observed fracture-free outcome is attributable to the anabolic action of romosozumab during the treatment phase vs the subsequent antiresorptive effect of zoledronic acid or denosumab during follow-up, particularly in a small case series without a control group. The favorable clinical trajectory likely reflects the combined and synergistic effects of both treatment phases.

Despite its retrospective design and small sample size, our study has several important strengths. First, it represents, to our knowledge, the first case series to systematically document clinical outcomes with romosozumab specifically in patients with established RVFs following denosumab discontinuation, extending beyond the isolated case reports previously published.^6^^,^^7^ Second, it includes microarchitectural data assessed by HR-pQCT, offering insights beyond standard densitometry. Third, all patients were followed prospectively for new clinical fractures, with systematic and log-term monitoring of treatment response.

In conclusion, romosozumab was effective in suppressing bone turnover and preventing new clinical fractures in these patients with RVFs following denosumab discontinuation. These findings suggest that romosozumab may be a viable therapeutic option for selected patients in this high-risk scenario. Prospective studies with larger cohorts are needed to confirm these results and clarify its role in clinical practice.

## Data Availability

The data that support the findings of this study are not publicly available due to privacy concerns, but may be made available from the corresponding author upon reasonable request.
